# A graph attention–based deep learning network for predicting biotech–small-molecule drug interactions

**DOI:** 10.1093/bioadv/vbaf192

**Published:** 2025-09-01

**Authors:** Fatemeh Nasiri, Mohsen Hooshmand

**Affiliations:** Department of Computer Science and Information Technology, Institute for Advanced Studies in Basic Sciences (IASBS), Zanjan, 45137-66731, Iran; Department of Computer Science and Information Technology, Institute for Advanced Studies in Basic Sciences (IASBS), Zanjan, 45137-66731, Iran

## Abstract

**Motivation:**

The increasing demand for effective drug combinations has made drug-drug interaction prediction a critical task in modern pharmacology. While most existing research focuses on small-molecule drugs, the role of biotech drugs in complex disease treatments remains relatively unexplored. Biotech drugs, derived from biological sources, have unique molecular structures that differ significantly from those of small molecules, making their interactions more challenging to predict.

**Results:**

This study introduces a novel graph attention network**–**based deep learning framework that improves interaction prediction between biotech and small-molecule drugs. Experimental results demonstrate that the proposed method outperforms existing methods in multiclass drug-drug interaction prediction, achieving superior performance across various evaluation types, including micro, macro, and weighted assessments. These findings highlight the potential of deep learning and graph-based models in uncovering novel interactions between biotech and small-molecule drugs, paving the way for more effective combination therapies in drug discovery.

**Availability and implementation:**

The datasets and source code of this study are available in the GitHub repository: https://github.com/BioinformaticsIASBS/BSI-Net.

## 1 Introduction 

### 1.1 Background

The increasing prevalence of complex diseases, particularly among the elderly, often necessitates the use of multiple medications. While polypharmacy aims to address different aspects of disease management and can lead to synergistic effects [e.g. aspirin for blood thinning with clopidogrel for preventing clot formation in patients with cardiovascular problems (Steinhubl *et al.* 2009)], it also increases the risk of unexpected drug-drug interactions (DDIs) and adverse side effects (Deng *et al.* 2020). Consequently, regulatory and monitoring institutions have developed various *in silico*, *in vitro*, *preclinical*, and *clinical* methods to identify potential DDIs and inform patients about their associated risks (Erceg and Antolović Amidžić 2025).

Monotherapy is often inadequate for effective treatment, as managing complex diseases typically requires drug combinations. Consequently, timely and costly clinical trials are necessary to ensure minimal side effects, a key focus of DDI research. Deep learning methods offer the potential to reduce these expenses through computational prediction of DDIs (Torkamannia *et al.* 2023, Xia *et al.* 2025).

Due to their pharmacological and pharmacokinetic properties, small-molecule drugs play a critical role in the treatment of diseases and the discovery of new drugs. Advances in deep learning methods have significantly accelerated the discovery of new small-molecule drugs, which now account for approximately 98% of all drugs (Li *et al.* 2025). As a result, DDI prediction for small-molecule drugs has also gained considerable attention. A plethora of research studies have utilized computational methods for DDI prediction. HDN-DDI (Sun and Zheng 2025), SSF-DDI (Zhu *et al.* 2024), DDI-Transform (Su and Qian 2024), BINDTI (Peng *et al.* 2025), and SumGNN (Yu *et al.* (2021) are graph neural network (GNN)-based methods that use various GNN architectures to model molecular structures and interactions. MCNN-DDI (Asfand-E-Yar *et al.* 2024), CNN-DDI (Zhang *et al.* 2022), and CNN-Siam (Yang *et al.* 2023) utilized CNN-based architectures to extract features from drug sequences, proteins, or other drug-related data such as Simplified Molecular Input Line Entry System (SMILES) and targets.

Although most studies have focused on small-molecule drugs, it is essential not to overlook biotech drugs in health and medicine. Biotech drugs have proven highly effective in treating diseases with high morbidity and mortality, such as cancer (Asadipour *et al.* 2023). For example, insulin, a biotech drug, is essential for individuals with diabetes (Ngo and Garneau-Tsodikova 2018). Therefore, it is crucial to emphasize that both small-molecule and biotech drugs warrant further investigation and exploration in drug discovery research. Small-molecule drugs have simple, well-defined chemical structures that can be represented using SMILES notation, making them easier to synthesize and modify. In contrast, biotech drugs are larger, protein-based molecules with complex structures that cannot be easily represented by SMILES. For example, Aspirin (Fig. S1, available as supplementary data at *Bioinformatics Advances* online) is a small-molecule drug with a well-defined SMILES representation, whereas Insulin Lispro (Fig. S2, available as supplementary data at *Bioinformatics Advances* online) is a biotech drug with a complex amino acid sequence that cannot be easily represented by SMILES (Wishart *et al.* 2018). Predicting interactions between biotech drugs and small molecules (BSI) presents distinct challenges compared to conventional DDI. First, the structural complexity of biotech drugs—large, heterogeneous molecules with post-translational modifications—makes computational modeling of their binding mechanisms difficult (Gayathiri *et al.* 2023). Second, immunogenicity introduces unpredictability, as biologic-induced immune responses can indirectly alter the pharmacokinetics or pharmacodynamics of co-administered small molecules (Jawa *et al.* 2013). Third, target specificity differs from small-molecule mechanisms, as biotechs often act via receptor binding or immune modulation rather than enzymatic inhibition, necessitating novel prediction approaches (Ryman and Meibohm 2017). Additionally, limited data availability biases current public DDI databases (e.g. DrugBank, TwoSIDES database as a resource of polypharmacy side effects for pairs of drugs) toward small molecules, leaving BSIs sparsely annotated (Wishart *et al.* 2018). Finally, biotech drugs can behave in complicated ways inside the body. They are not always eliminated at a constant rate, and their mechanisms depend on target binding, making dose–response predictions challenging (Mager and Jusko 2001).

Additionally, they exhibit distinct behaviors in absorption, distribution, metabolism, and excretion within the body (Poduval *et al.* 2023). Together, these challenges underscore the need for specialized methods to study BSIs, making research on interactions between these drug types crucial for advancing drug discovery.

This work proposes a graph-attention–based method with deep models that utilizes the similarity and sequence properties, as well as the domain-specific language model methods of small-molecules and biotech drugs to predict their interactions. To do this, we generated and proposed a new dataset that contains the last approved drugs from the DrugBank, with their related and significant relation with DDI prediction.

The structure of this article is as follows. Section 1.2 introduces the related work briefly. Then, Section 2.1 introduces the generated data proposed in this work. Section 2.2 proposes the BSI-Net method for biotech–small-molecule DDI prediction. Section 3 reports the results and Section 4 reports the findings about critical DDIs. Finally, Section 5 concludes the paper.

### 1.2 Related work

As mentioned earlier, there exists a plethora of research studies focused on small-molecule DDI prediction. Some of them have been briefly introduced in the Supplementary Information, available as supplementary data at *Bioinformatics Advances* online. However, only a limited number of studies propose methods for biotech–small-molecule pair DDI prediction. We briefly describe these types of biotech–small-molecule DDI prediction studies below.

Huang *et al.* (2022) proposed a three-step method for predicting interactions between small-molecules and biotech drugs. In the first step, they utilized different feature representations of small-molecules and biotech drugs (e.g. structural and interactive properties) and applied operations such as Jaccard similarity computation and principle component analysis (PCA) to generate feature vectors. However, as our assessment and confirmation in Ru *et al.* (2023) indicate, the authors did not compute one-hot encodings correctly and failed to perform the Jaccard computation accurately. They applied PCA directly on the incorrect one-hot encodings. Additionally, they employed a proposed type of negative sampling. The final step involved using a deep learning model to predict the types of interactions. However, they reported performance based on a dichotomization of negative and positive data and did not provide the results for the correct prediction of interaction types.

Ru *et al.* (2023) utilized a two-level graph representation: one level for capturing DDIs and DTIs and another for graph entities corresponding to each small-molecule drug. They applied a type of graph convolutional network to generate embeddings and subsequently used a multilayer perceptron (MLP) to predict interaction types between biotech–small-molecule pairs. However, they reported results primarily for small-molecule DDIs, which do not directly align with their main objective.

Some biotech drugs have multiple sequences listed in DrugBank. Often, the first sequence is identical across several drugs, while variations occur in the subsequent sequences. For example, Insulin aspart, Insulin detemir, and Insulin glulisine all share the same first sequence, but differ in the second sequence. Based on the assessment of Huang *et al.* (2022) and Ru *et al.* (2023), both studies consider only the first sequence for downstream tasks. In contrast, this work utilizes all sequences of a drug for downstream tasks.

In another study, Nasiri and Hooshmand (2024), the authors explored machine and deep learning methods for predicting biotech–small-molecule DDIs. They applied machine learning to structural and interaction properties of the drugs to perform binary prediction. Their results indicated that CNN achieved the best performance for binary prediction. However, this work demonstrates that CNN is not the optimal choice for multiclass prediction. Furthermore, this work distinguishes itself in two key ways: (i) the introduction of a new dataset and (ii) the proposal of a novel method.

## 2 Methods 

### 2.1 Dataset

To propose a new method for the prediction of the interaction between biotech-type drugs and small-molecule-type drugs, we report our exploration of the literature. Huang *et al.* (2022) proposed a dataset that contains information on those two types of drugs. However, the number of biotech drugs was not enough for further analysis. Ru *et al.* (2023) proposed two different datasets, one for predicting biotech–small-molecule interactions and the other is just for small-molecule DDI predictions. The authors mentioned the problems of the former method and the dataset. However, their dataset suffers from the same problem as the former. Their dataset has only the information on 55 biotech drugs. Although this work has the aim of proposing a new learning model to improve the prediction performance, it introduces a new dataset of information on these two types of drugs. Thus, this section describes the proposed dataset.

All the requirements for dataset generation were collected from DrugBank (Wishart *et al.* 2018). The dataset contains information on three identities, i.e. Biotech drugs (protein-based), small-molecule drugs (chemical drugs), and proteins or targets with which the drugs interact. While the total number of reported biotech drugs in the DrugBank was 1620, those drugs of this type which has been approved and their corresponding sequences are available are 196. Therefore, we collected the available information on the number of biotech drugs. Furthermore, the number of small-molecule drugs that were approved and their SMILES were available was equal to 2148. Thus, we collected their name and SMILES from the DrugBank. The total number of targets was 2186. But the unique number of total drugs (both types) that had an interaction with any protein was 1953. In other words, there existed drugs with no interaction with any target.

It is worth mentioning that there are two general types of interactions—DDI and drug-target interactions (DTI). DDIs themselves are divided into three subtypes of biotech-biotech interactions (BBI), small-molecule—small-molecule interactions (SSI), biotech–small-molecule interactions (BSI). This work considers BSIs as the labels and aims to predict their values efficiently and effectively. There are 5034 BBIs, 612 947 SSIs, and 45 205 BSIs, therefore, the total is 663 186 interactions. The total number of DTIs is 7814. DTIs are divided into two types of biotech-target interactions (BTI) and small-molecule-target interactions (STI). There are 409 and 7405 of BTIs and STIs, respectively. The total number of unique targets that play a role in BTIs and STIs is 197 and 2056, respectively. Table 1 provides an overview of biotech and small-molecule drugs, including their targets and interactions.

**Table 1.** Statistics of drugs, targets, and interactions in the dataset.

Entity	Biotech drugs	small-molecule drugs
Count	196	2148
Unique targets	197	2056
DTI	409	7405

As mentioned above, the BSIs are considered as labels. We consider these labels as the positive labels which are equal to 45 205. These positive labels are sentences that reports the interaction between each BSI pair. Figure S3, available as supplementary data at *Bioinformatics Advances* online represents some examples of such reports. We consider each of them as a separate label. There are 96 types of positive labels, with the frequencies of the labels ranging from 1 to 8809.

There are 31 BSI labels with frequencies above 100 (Table S1, available as supplementary data at *Bioinformatics Advances* online shows the statistics of the labels in descending order sorted). Therefore, we keep these as the number of positive types of labels, considering that the total number of existing BSIs is 43 876. Having this primary raw information, we have prepared the dataset as follows.

- small-molecule drugs have SMILES and have interactions with targets. From these values, we computed four types of feature vectors for small-molecule drugs.
  - We generated an interaction matrix for small-molecule drugs, resulting in a 2148×2148 matrix called “${\text{SS}}_{I}$.”
  - We used Tanimoto score (Bajusz *et al.* 2015) to compute the similarity among them. As mentioned earlier, there are 2148 drugs of type small-molecule in the dataset. Therefore, we use the similarity values to generate a feature vector for each drug with size 2148. We call this feature matrix “${\text{SS}}_{s}$.” Its dimension is 2148×2148.
  - Then we generated the “STI” matrix (size 2148 × 2056), which represents the interactions between small-molecule drugs and targets. Then, we apply cosine similarity on the vectors within the “STI” matrix to calculate small-molecule similarities based on their interactions with the targets (small-molecule interaction-derived similarity matrix), ${\text{SS}}_{T}$, with a size of 2148×2148.
  - In addition to similarity checking, we generated bidirected DGL graphs for each drug from its SMILES representation using DGL-LifeSci (Li *et al.* 2021).
- The biotech drugs have sequences and known interactions with targets. From these, we derived multiple types of feature vectors.
  - We generated an interaction matrix for biotech drugs, resulting in a 196×196 matrix called “${\text{BB}}_{I}$.”
  - We computed a similarity matrix for biotech drugs based on their interactions with targets using cosine similarity. Notably, the set of 197 targets interacting with biotech drugs differs from those associated with small-molecules. Therefore, we construct the “BTI” matrix (size 196×197) to represent these interactions. Using cosine similarity, we then generated the biotech drug similarity matrix (biotech interaction-derived similarity matrix), called “${\text{BB}}_{T}$,” with a size of 196×196.
  - We utilized protein drug sequences and applied the ProtBert model to generate embedding vectors of size 1024 for these drugs. ProtBert is a pretrained language model based on the BERT architecture and Transformers, specifically designed for processing and analyzing protein sequences. By leveraging deep learning on a vast dataset of protein sequences, ProtBert can capture complex relationships between amino acids and extract biologically meaningful features from raw sequences Brandes *et al.* (2022). For drugs with multiple sequence chains, we first computed separate embedding vectors for each chain and then averaged them to obtain a single embedding vector per drug. The resulting embeddings form a matrix called “$B_{\text{ProtB}}$,” with dimensions 196×196.

Table 2 shows the summary of the generated features in the proposed dataset.

**Table 2.** Overview of feature representations in the proposed dataset.

Drug type	Feature representation	Dimensions
Small-molecule	Small-molecule drug-drug interaction matrix (${\text{SS}}_{I}$)	2148×2148
	Structural similarity matrix (Tanimoto, ${\text{SS}}_{s}$)	2148×2148
	Small-molecule interaction-derived similarity matrix (${\text{SS}}_{T}$)	2148×2148
	Graph-based representation (DGL)	–
Biotech	Biotech drug-drug interaction matrix (${\text{BB}}_{I}$)	196×196
	Sequence similarity matrix (Global alignment, ${\text{BB}}_{s}$)	196×196
	Biotech interaction-derived similarity matrix (${\text{BB}}_{T}$)	196×196
	Protein sequence embeddings using ProtBert($B_{\text{ProtB}}$)	196×1024

### 2.2 Proposed method

This section proposes BSI-Net, a new graph attention-based method for BSI predictions. Before delving into the proposed method, we mention the inputs and basis methods that we have examined in order to compare the BSI-Net performance.

From the BSI-data, we construct feature vectors for small-molecules and biotech drugs as follows. We use ${\text{SS}}_{I}$, ${\text{SS}}_{T}$, and ${\text{SS}}_{s}$ for small-molecule feature vectors in such a way that the input is their summation ${\text{SS}}_{f}{=\text{SS}}_{I}{+\text{SS}}_{T}{+\text{SS}}_{s}$, and for biotech drugs we use the summations of ${\text{BB}}_{I}$, ${\text{BB}}_{T}$, and ${\text{BB}}_{s}$, i.e. ${\text{BB}}_{f}{=\text{BB}}_{I}{+\text{BB}}_{T}{+\text{BB}}_{s}$ as their input vectors. To predict the interaction type between the *i*th small-molecule and *j*th biotech drug, our proposal concatenates the *i*th row of ${\text{SS}}_{f}$ with the *j*th row of ${\text{BB}}_{f}$, forming the input vector for classification. We evaluate our approach using three machine learning models, including support vector machine (SVM), random forest (RF), and XGBoost. SVM is a powerful machine learning model useful in classification and regression that looks for a hyperplane with the maximum distance from the samples of the two categories (Wang 2002). The random forest is another high-performance machine learning model that uses a set of decision trees, each a subsample of features. Each tree decides whether the category is the input to the prediction models. The final choice for the random forest is the majority vote, which conceptually is based on the use of several weaker learning models to avoid overfitting in the final learning (Breiman 2001). XGBoost is a fast and highly efficient machine learning algorithm that is based on gradient boosting, which in each iteration creates a new tree to reduce the construction errors of the previous tree. Furthermore, it assigns a larger weight to the samples that caused the error in the previous trees. This property equips the XGBoost learning model more efficiently. In the final step, all the trees are combined to produce a powerful learning model (Chen and Guestrin 2016).

#### 2.2.1 Graph attentio network

BSI-Net utilizes a graph attention network (GAT) to have more significant embeddings. Thus, we describe the attention model before describing the proposed method. Given a graph G=(V,E), where *V* is the set of nodes and *E* is the set of edges, the GAT updates the node embeddings using attention mechanisms (Veličković *et al.* 2017). The first step is feature transformation, in which, each node *i* has an input feature vector $h_{i}$. The GAT first applies a linear transformation as follows;

(1) $$h_{i}^{\left(\text{trans}\right)}=Wh_{i},$$

where W is a learnable weight matrix. Then, for each edge between nodes *i* and *j*, we calculate an attention score using the following equation;

(2) $$e_{ij}=\text{LeakyReLU}\left(a^{T}\left[Wh_{i}\|Wh_{j}\right]\right),$$

where a is a learnable attention vector, ‖ denotes concatenation, LeakyReLU is the activation function. Afterwards, the attention coefficients are computed. In other words, attention scores are normalized using the softmax function as follows;

(3) $$\alpha_{ij}=\frac{\;\text{exp}(e_{ij})}{\sum_{k\in N(i)}\;\text{exp}\;(e_{ik})},$$

where N(i) is the set of neighbors of node *i*. The new representation of node *i* is calculated by aggregation of the feature vectors of its neighbors;

(4) $$h_{i}^{\prime }=\sigma \left(\sum_{j\in N(i)}\alpha_{ij}Wh_{j}\right),$$

where σ is a non-linear activation function, e.g. ReLU. For stability and efficient learning, multiple attention heads are used, as follows;

(5) $$h_{i}^{\left(\text{head}\;k\right)}=\sum_{j\in N(i)}\alpha_{ij}^{\left(k\right)}W^{\left(k\right)}h_{j}$$

The final node representation is computed by either averaging,

(6) $$h_{i}^{\prime }=\frac{1}{K}\sum_{k=1}^{K}\left(\sum_{j\in N(i)}\alpha_{ij}^{\left(k\right)}h_{j}^{\left(k\right)}\right)$$

or concatenating the outputs of multiple heads.

(7) $$h_{i}^{\prime }={\|}_{k=1}^{K}\left(\sum_{j\in N(i)}\alpha_{ij}^{\left(k\right)}h_{j}^{\left(k\right)}\right).$$

BSI-Net concatenates the outputs of multiple attention heads to form the final node representation.

#### 2.2.2 BSI-Net

**Figure 1.**
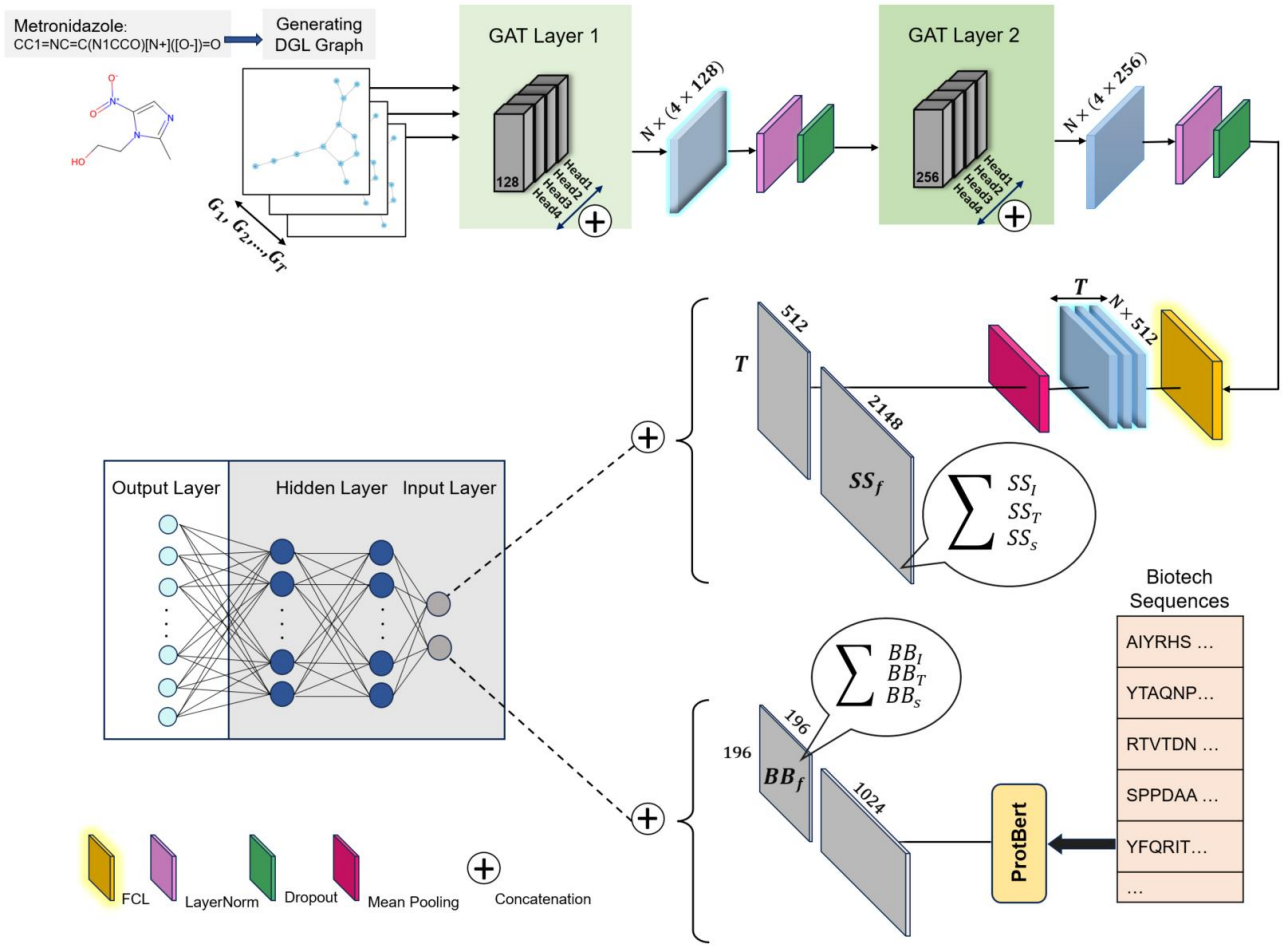
The BSI-Net framework predicts DDIs using small-molecule and biotech drug properties. For small-molecules, it converts SMILES strings to graphs using DGL, applies two GAT layers to generated representations, and concatenates these with similarity information (sum of ${\text{SS}}_{I}$, ${\text{SS}}_{T}$, ${\text{SS}}_{S}$). For biotech drugs, it uses ProtBert to create sequence representations, which are then concatenated with similarity features. Finally, BSI-Net combines the small-molecule and biotech drug representations and inputs them to an MLP to predict interaction types.

We have introduced the main components of our proposal, BSI-Net. This section describes the details of the framework. Figure 1 illustrates the general structure of BSI-Net. The inputs are categorized into two types: small-molecule drug properties and biotech drug properties.

For small-molecule properties, BSI-Net categorizes and processes them in two ways. The first type of property is derived from the SMILES representation of the drugs. BSI-Net converts these SMILES into graphs using deep graph library (DGL). It then applies a multihead graph attention layer with a size of 128 to each graph, utilizing four multihead attention mechanisms in this layer. The output for each node in the graph (N nodes in total) results in a matrix of size 4×128. Following this, BSI-Net applies normalization and a dropout layer with a drop rate of 0.2 to the node features. A second multihead graph attention layer, this time with a size of 256, is then applied, followed by another normalization layer and dropout layer. The processed data is then fed into a fully connected layer. Finally, a mean pooling layer is applied, resulting in feature vectors of size 512 for the small-molecule drugs.

The additional properties of the small-molecules are derived from similarity matrices. The summed results of ${\text{SS}}_{I}$, ${\text{SS}}_{T}$, and ${\text{SS}}_{S}$ yield ${\text{SS}}_{f}{=\text{SS}}_{I}{+\text{SS}}_{T}{+\text{SS}}_{S}$, which constitutes the next component of the small-molecule feature vectors, with a feature size of 2148. The output from the graph attention layer is concatenated with ${\text{SS}}_{f}$, giving a final feature vector size of 2660 for small-molecule drugs.

The biotech properties are further divided into two subsets. One subset is derived from the summation of the similarity matrices: ${\text{BB}}_{f}{=\text{BB}}_{I}{+\text{BB}}_{T}{+\text{BB}}_{S}$ with a feature size of 196. Additionally, the biotech sequences are processed through the pre-trained ProtBERT, resulting in feature vectors of size 1024. The final feature vectors for biotech drugs are produced by concatenating the results from ProtBERT ($B_{\text{ProtB}}$) and ${\text{BB}}_{f}$, leading to feature vectors with a size of 1220.

The previous steps lead to generating embeddings for small-molecules and biotech drugs. The final feature vector for DDI prediction is the concatenation of each small-molecule drug feature vector with the biotech feature vector. These final vectors are the inputs for the prediction part. BSI-Net uses an MLP for multiclass prediction. The network contains two hidden layers. The input layer has a size of 3880, which consists of concatenated embeddings from the small-molecule and biotech drug feature vectors. The output layer contains 32 neurons, corresponding to the number of interaction classes. The activation function in the hidden layers is ReLU, while the output layer uses the softmax function to predict the interaction class probabilities. BSI-Net employs the cross-entropy loss function to compute the prediction error, as defined in Equation (8). The model is optimized using the Adam optimizer, and hyperparameter tuning is performed via grid search with various configurations of hidden layer sizes, dropout rates, and learning rates.

(8) $$\text{Loss}=-\sum_{i=1}^{C}y_{i}\;\text{log}(p_{i})$$

The steps for performing BSI-Net are outlined in Algorithm 1. The algorithm requires several inputs: SMILES, the feature set of small-molecules (${\text{SS}}_{f}$), sequences of biotech drugs (${\text{BB}}_{f}$), labels (Y), and an error threshold (ϵ). The input data is divided into multiple folds. In each fold, the training phase continues as long as the training error exceeds the predefined threshold, as detailed in lines 4 to 14.

During the training phase, the SMILES data from the training subset (${\text{SMILES}}^{\left(\backslash k\right)}$) are processed using DGL to create small-molecule graphs, as shown in line 5. Line 6 applies the proposed GAT model to the output from DGL. The algorithm then concatenates the feature set of the training subset, ${\text{SS}}_{f}^{\left(\backslash k\right)}$, resulting in a feature vector for the small-molecules, denoted as $S_{T}$.

Algorithm 1 BSI-Net

1: **Input:** SMILES, ${\text{SS}}_{f}$, Seq, ${\text{BB}}_{f}$, *Y*, ϵ

2: **Output:**  $\hat{Y}$

3: **for** *k* in *k*-fold **do**

4:  **while**  E≥ϵ  **do**

5:    $S_{g}\leftarrow \text{DGL}({\text{SMILES}}^{\left(\backslash k\right)})$

6:    $S_{\text{GAT}}\leftarrow \text{GAT}(S_{g}^{\left(\backslash k\right)})$

7:    $S_{T}\leftarrow \text{Concate}({\text{SS}}_{f}^{\left(\backslash k\right)},S_{\text{GAT}})$

8:    $B_{\text{pBert}}\leftarrow \text{ProtBert}({\text{Seq}}^{\left(\backslash k\right)})$

9:    $B_{T}\leftarrow \text{Concate}({\text{BB}}_{f}^{\left(\backslash k\right)},B_{\text{pBert}})$

10:    $X^{\left(\backslash k\right)}\leftarrow \text{Concate}(S_{T},B_{T})$

11:    ${\hat{Y}}^{\left(\backslash k\right)}\leftarrow \text{MLP}(X^{\left(\backslash k\right)})$

12:    $E\leftarrow \text{Loss}(Y^{\left(\backslash k\right)},{\hat{Y}}^{\left(\backslash k\right)})$ using Equation (8)

13:    Backpropagation(E,AdamOptimizer)

14:   **end while**

15:   ${\hat{Y}}^{\left(k\right)}\leftarrow \text{EvaluateModel}(X^{\left(k\right)})$

16:   $\hat{Y}.\text{append}({\hat{Y}}^{\left(k\right)})$

17: **end for**

18: **Evaluate Model Performance:**

19:$\text{evaluation}\_\text{results}\leftarrow \text{EvaluateModel}(\hat{\text{Y}},Y)$

In lines 8 and 9, the feature vectors for biotech drugs are prepared. Line 10 concatenates the feature vectors of small-molecules and biotech drugs. Line 11 applies a MLP model to the combined feature vector to evaluate the interactions among drugs during the training phase. Line 12 calculates the loss for the predicted training values, following the loss function defined in Equation (8). Based on this calculated error, the network weights are updated through backpropagation in line 13.

Once the training subset converges, the labels for the test subset are computed. This process is repeated for all folds. Finally, the evaluation results are compiled in line 19.

## 3 Results

We employ a k-fold stratified cross-validation approach to evaluate the performance of BSI-Net. The stratified division ensures that each fold maintains the same label distribution as the overall dataset, which is particularly important for handling imbalanced labels. Consequently, both the training and test sets contain a proportional representation of each label (Prusty *et al.* 2022).

As mentioned earlier, our dataset consists of 31 positive labels and one negative label, totaling 32 labels. To assess model performance comprehensively, we report results based on three categories of evaluation metrics: micro, macro, and weighted. These metrics are particularly useful when dealing with multiclass classification tasks (Grandini *et al.* 2020).

This research reports the evaluation metrics for method comparison in three different regime of micro, macro, and weighted. Their full description is in Section S5 of the Supplementary Information, available as supplementary data at *Bioinformatics Advances* online.

To ensure a comprehensive performance evaluation, we compare the results of the mentioned random forest, SVM, and XGBoost models. Additionally, this study implemented a three-layer MLP for comparison. Furthermore, this work utilizes the CNN architecture introduced in Hashemi *et al.* (2023) and Zabihian *et al.* (2023), which consists of three layers. Each layer includes a convolutional layer, batch normalization, and a dropout mechanism. Following these layers, the architecture incorporates a dense layer and concludes with an activation layer. Finally, we compare the performance of BSI-Net with the results reported in Huang *et al.* (2022); Ru *et al.* (2023). Figure S4, available as supplementary data at *Bioinformatics Advances* online shows the confusion matrix of the predicted labels against the true labels of 32 labels (31 positive labels plus the negative label).

### 3.1 Micro and macro

Table 3 reports the results based on micro performance for Matthews Correlation Coefficient (MCC), Area Under the Receiver Operating Characteristic Curve (AUROC), and Area Under the Precision-Recall (AUPR) metrics. BSI-Net exhibits the best performance except in AUPR, where it ranks second, and random forest takes the top position. This underscores the necessity for researchers to consider the strength of machine learning methods when proposing techniques. It is worth mentioning that Table S4, available as supplementary data at *Bioinformatics Advances* online shows the micro results based on accuracy, precision, recall, and F1 score. While the values in each row are equal, this observation is typical in micro value computations, making it less favorable for evaluating performance.

**Table 3.** Evaluation of methods in micro regime.

Methods	MCC (std)	AUROC (std)	AUPR (std)
SVM	0.9675 (0.002))	0.9997 (0.000)	0.9946 (0.000)
RF	0.9761 (0.002)	0.9997 (0.000)	**0.9974 (0.000)**
XGBoost	0.9625 (0.002)	0.9998 (0.000)	0.9953 (0.001)
MLP	0.6941 (0.013)	0.9877 (0.001)	0.7980 (0.014)
CNN	0.5629 (0.040)	0.9781 (0.004)	0.6621 (0.053)
BSI-Net	**0.9809** (**0.012)**	**1.0000** (**0.000)**	0.9908 (0.009)

Bold values indicate the best performance.

Table 4 presents the evaluation metrics in the macro regime. BSI-Net shows the best performance across all metrics, while XGBoost takes the next rank. CNN has the lowest performance; in fact, all models except CNN achieve AUPR values above 0.9.

**Table 4.** Evaluation of methods in macro regime.

Methods	Precision (std)	Recall (std)	F1-score (std)	AUROC (std)	AUPR (std)
SVM	0.9649 (0.002)	0.9562 (0.006)	0.9598 (0.003)	0.9989 (0.000)	0.9783 (0.004)
RF	0.9775 (0.004)	0.9510 (0.006)	0.9614 (0.006)	0.9986 (0.001)	0.9831 (0.005)
XGBoost	0.9721 (0.004)	0.9410 (0.003)	0.9532 (0.002)	0.9990 (0.000)	0.9837 (0.002)
MLP	0.5632 (0.020)	0.9228 (0.004)	0.6551 (0.018)	0.9933 (0.001)	0.9040 (0.005)
CNN	0.4677 (0.026)	0.8619 (0.010)	0.5476 (0.029)	0.9855 (0.002)	0.8007 (0.014)
BSI-Net	**0.9705 (0.053)**	**0.9609 (0.073)**	**0.9627 (0.058)**	**0.9999 (0.000)**	**0.9908 (0.026)**

Bold values indicate the best performance.

### 3.2 Weighted

In addition to micro and macro averages, weighted evaluation metrics were computed (Table 5). BSI-Net performs best, followed by random forest, while CNN performs worst. Considering all three evaluation modes (micro, macro, and weighted), BSI-Net achieves the highest performance, followed by random forest and XGBoost. MLP and CNN exhibit the lowest performance, with MLP outperforming CNN. Furthermore, the results for the macro regime are reported in Supplementary Information, available as supplementary data at *Bioinformatics Advances* online.

**Table 5.** Evaluation of methods in weighted regime.

Methods	Precision (std)	Recall (std)	F1-score (std)	AUROC (std)	AUPR (std)
SVM	0.9770 (0.001)	0.9764 (0.001)	0.9765 (0.001)	0.9986 (0.000)	0.9906 (0.001)
RF	0.9826 (0.001)	0.9827 (0.001)	0.9824 (0.001)	0.9995 (0.000)	0.9961 (0.000)
XGBoost	0.9729 (0.002)	0.9730 (0.002)	0.9724 (0.002)	0.9991 (0.000)	0.9935 (0.001)
MLP	0.8592 (0.007)	0.7381 (0.014)	0.7655 (0.014)	0.9788 (0.002)	0.9294 (0.006)
CNN	0.7981 (0.013)	0.6048 (0.048)	0.6367 (0.047)	0.9473 (0.009)	0.8540 (0.014)
BSI-Net	**0.9866 (0.022)**	**0.9861 (0.025)**	**0.9861 (0.021)**	**0.9999 (0.000)**	**0.9981 (0.007)**

Bold values indicate the best performance.

Table 6 compares BSI-Net’s performance against the state-of-the-art methods [multi-SBI (Huang *et al.* 2022) and CB-TIP (Ru *et al.* (2023)], but a direct comparison is limited due to varying numbers and types of drugs used, detailed in the table. BSI-Net and CB-TIP (Ru *et al.* 2023) results are weighted, while multi-SBI (Huang *et al.* 2022) reports binary (positive/negative) classification results instead of multiclass metrics. Furthermore, CB-TIP uses more small-molecules than BSI-Net, which exclusively considers approved drugs from the latest DrugBank list, unlike CB-TIP. For a fair comparison, we implemented and compared our BSI-Net against CB-TIP, the top method in the literature. As shown in Table S5, available as supplementary data at *Bioinformatics Advances* online, BSI-Net outperforms CB-TIB. An ablation study of BSI-Net’s modules, detailed in Section S6.2 of the Supplementary Information, available as supplementary data at *Bioinformatics Advances* online, confirms the method’s performance.

**Table 6.** BSI-Net outperforms state-of-the-art methods [multi-SBI (Huang *et al.* 2022) and CB-TIP (Ru *et al.* 2023)] in weighted biotech–small-molecule DDI prediction.

Method	Biotechs	Small-molecules	F1-score (std)	AUROC (std)	AUPRC (std)
Multi-SBI^a^	148	1941	0.8673	0.9997	0.9892
CB-TIP	55	3119	0.9780 ± 0.08	0.9666 ± 0.05	0.9420 ± 0.10
BSI-Net	196	2148	**0.9861** ± **0.021**	**0.9999** ± **0.000**	**0.9981** ± **0.007**

a Multi-SBI’s multiclass results were reported as a binary dichotomization, as they did not report a weighted version.

Bold values indicate the best performance.

**Figure 2.**
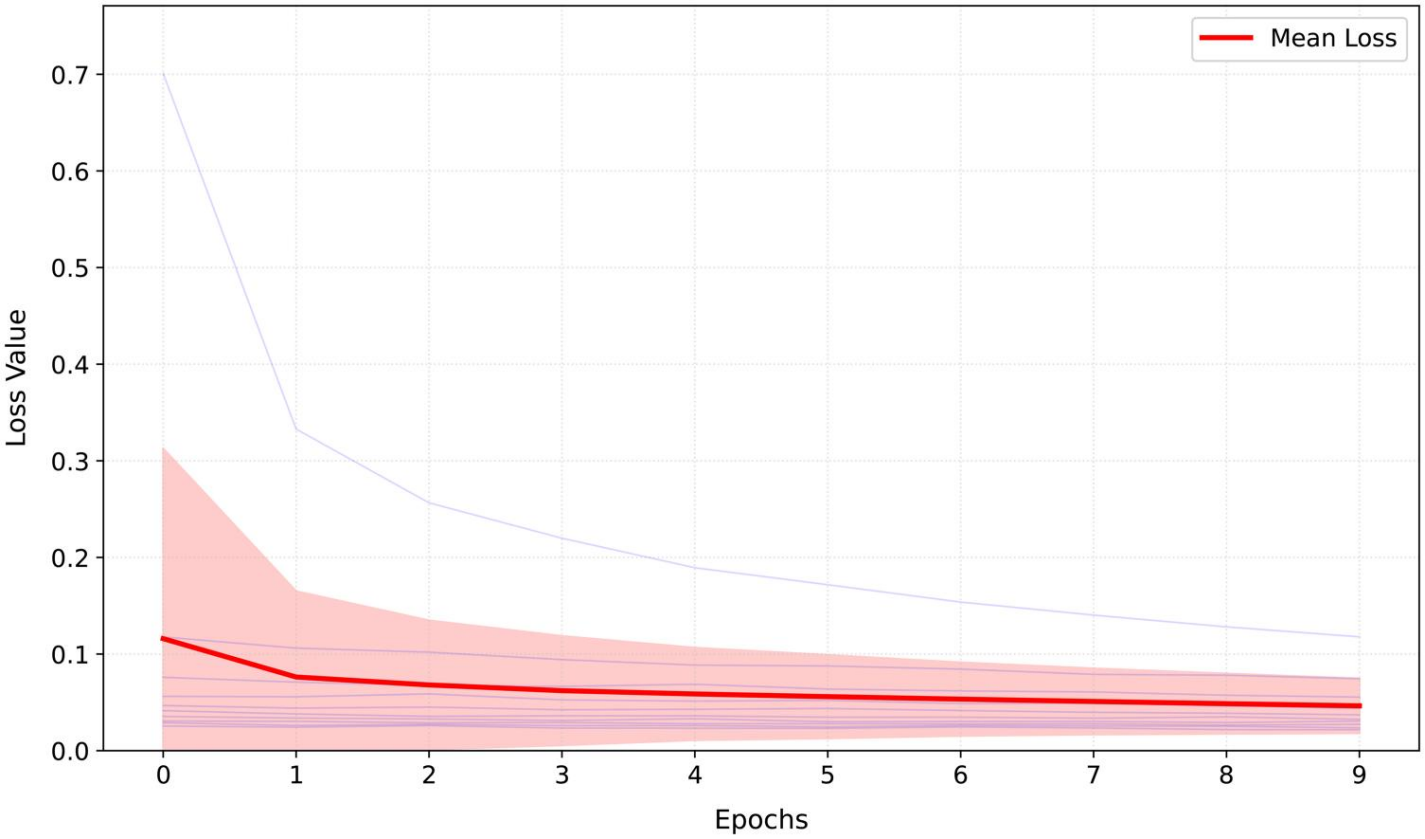
The figure displays all 10 training folds (semi-transparent blue lines), their mean trajectory (bold red line), and standard deviation bands (pink shading), ensuring complete transparency into the model’s convergence dynamics.

**Figure 3.**
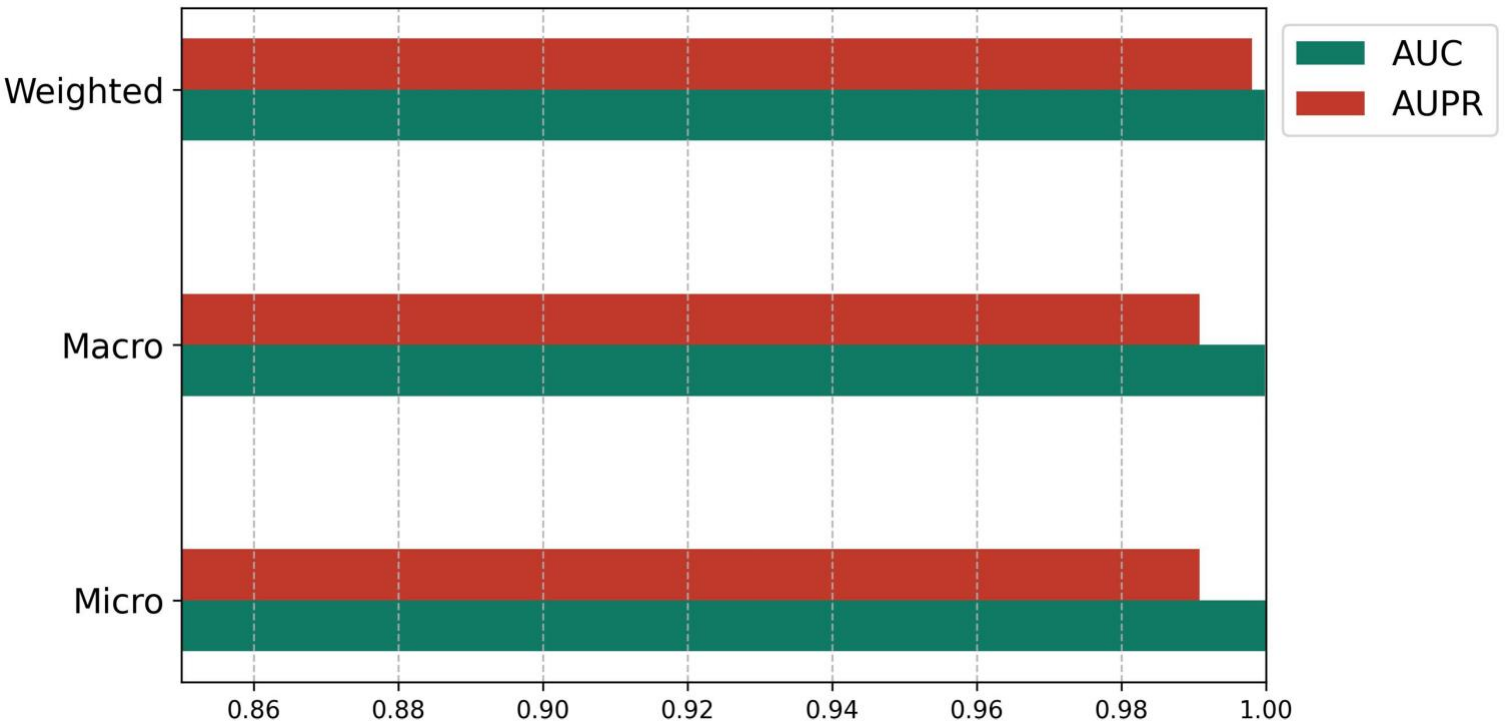
BSI-Net performance.

Figure 2 shows BSI-Net convergence in 10 epochs. The training loss curve exhibits a clear downward trend across epochs, indicating effective learning. The smooth convergence of the loss values—particularly as the curve stabilizes in later epochs (7-9)—suggests stable training without erratic fluctuations. Figure 3 presents AUROC and AUPR bar charts for BSI-Net in three modes.

Table S12, available as supplementary data at *Bioinformatics Advances* online presents a subset of biotech–small-molecule drug pairs along with their corresponding current interaction labels and the predicted labels generated by BSI-Net.

## 4 Discussion

Human, pork, and beef insulin are considered very similar because their core structure is almost identical (Blundell *et al.* 1972, Vecchio *et al.* 2018). All three are proteins made of two chains (A and B) linked together (Vecchio *et al.* 2018). Pork insulin differs from human insulin by only one building block (amino acid) out of 51. Beef insulin differs by three amino acids (Brange 2012, Vecchio *et al.* 2018). Crucially, these tiny differences are not in the parts of the insulin molecule most important for its job: lowering blood sugar (De Meyts 2016). Because the key working parts are the same, pork and beef insulin can bind to the human body’s insulin receptors and effectively signal cells to take up glucose, just like human insulin does (Kurtzhals *et al.* 2000). This is why insulin extracted from pigs and cows was successfully used to treat diabetes in millions of people for over 60 years before scientists could make perfect human insulin in labs (Johnson 1983, Schernthaner 1993). According to DrugBank (Wishart *et al.* 2018), the metabolism of praziquantel can be increased when combined with either pork or beef insulin (DrugBank Accession Numbers: DB01058 for praziquantel, DB00071 for insulin pork, DB09456 for insulin beef). This interaction likely occurs because insulin activates cellular uptake and metabolic pathways (e.g. via insulin receptor signaling) (Saltiel and Kahn 2001, De Meyts 2016), which may accelerate the clearance of praziquantel. Given the near-identical structure and function of human, pork, and beef insulin—where pork differs by only one amino acid (B30) and beef by three (A8, A10, B30), none of which affect critical receptor-binding regions (Brange 2012, Vecchio *et al.* 2018)—it is biologically plausible that human insulin would similarly enhance praziquantel metabolism. The conserved mechanism of action across all three insulin types (binding to the human insulin receptor and triggering downstream metabolic effects) (Blundell *et al.* 1972) supports extrapolating this interaction to human insulin. Thus, while DrugBank specifically lists pork and beef insulin, the interaction likely applies to human insulin as well, unless evidence suggests otherwise.

## 5 Conclusion

This work has presented a novel DDI prediction method for biotech–small-molecule drug pairs, leveraging GATs, domain-specific language model representations, and MLP analysis of drug properties. We have introduced a new dataset and demonstrated the superior performance of our approach. Furthermore, we have highlighted the relevance of comparing our method with lighter machine learning models, which can offer sufficient performance for drug interaction prediction in drug discovery. Our method currently relies on similarity vectors of the drugs, and future research will explore using direct data representations for potentially improved results. Improved sampling enhances performance. CNNs, effective for binary prediction, struggle with multiclass problems. Exploring anomaly detection as an alternative to negative sampling is crucial for better interaction prediction. Tables S6 and S7, available as supplementary data at *Bioinformatics Advances* online show the best-tuned hyperparameters of the implemented methods.

## Supplementary Material

vbaf192_Supplementary_Data

## Data Availability

The datasets and code generated in this study are available in the GitHub repository: https://github.com/BioinformaticsIASBS/BSI-Net.
