# Supplementary material for: A graph attention–based deep learning network for predicting biotech–small-molecule drug interactions
Source: Bioinform Adv. 2025 Sep 1;5(1):vbaf192. doi: 10.1093/bioadv/vbaf192 (PMC12408249; doi:10.1093/bioadv/vbaf192)
Supplement: vbaf192_Supplementary_Data [file vbaf192_supplementary_data.pdf]

Supplementary Information

A Graph-Attention-Based Deep Learning Network for Predicting Biotech–Small-Molecule Drug Interactions

Fatemeh Nasiri[1], Mohsen Hooshmand[1]

[1]Department of Computer Science and Information Technology, Zanjan, Iran

© Email: mohsen.hooshmand@iasbs.ac.ir

1 Small Molecule vs. Biotech

Figure 1 and Figure 2 demonstrate the structure of Aspirin as a Small molecule and Insulin Lispro as a Biotech drug Wishart et al. [2018].

Figure 1: Structure of Aspirin, a small-molecule drug with its atomic arrangement and bonds.

Figure 2: Structure of Insulin Lispro, a biotech drug with its modified protein sequence.

2 Related work

In the field of drug-drug interaction prediction, machine learning methods can be divided into six categories: Similarity-based approaches, Traditional classification, Network diffusion, Matrix factorization, Ensemble-based approaches, and literature-based Han et al. [2022]. To predict conventional and synthetic DDIs, Yu et al. [2018] developed a DDINMF approach based on semi-non-negative matrix factorization. Using drug features and known DDI information, Zhang et al. [2017] proposed a prediction model combining neighbor-recommendation, random walk, and matrix perturbation strategies in a versatile ensemble framework.

Ryu et al. [2018] introduced DeepDDI, a deep learning framework that predicts drug-drug and drug-food interactions using compound names and SMILES-based structural similarity profiles. The framework converts molecular structures into feature vectors and processes them through a multi-label neural network to identify possible interaction types. Yan et al. [2020] introduced DDI-IS-SL, a method for predicting drug-drug interactions by combining different types of drug similarity. This method calculates similarities using chemical, biological, and phenotypic data, incorporates additional similarity measures using a Gaussian kernel, and then applies a machine learning model to predict interaction scores. Zhu et al. [2020] introduced DGDFS, a feature selection method for predicting adverse DDIs. This method uses dependency-guided learning to select features based on their interdependencies, focusing on feature relationships within large datasets. Chen et al. [2021] proposed MUFFIN, a multi-scale feature fusion framework for DDI prediction. The framework integrates features from different scales to capture complex relationships between drugs, utilizing both molecular and network-level information.

Lin et al. [2022] developed MDF-SA-DDI, a deep learning model that predicts drug-drug interactions using multiple types of drug information based on similarity. The model employs neural networks to extract features from the data and utilizes a transformer architecture to integrate them for improved predictions. Yu et al. [2022] proposed RANEDDI, a model for predicting DDIs using relation-aware network embedding. This approach captures both the structural features of drug networks and the specific types of relationships between drugs to generate more accurate interaction predictions. Al-Rabeah and Lakizadeh Al-Rabeah and Lakizadeh [2022] proposed a graph neural network approach for predicting DDIs and their associated effects. Their model constructs a network representation of drugs from various data sources, generates drug embeddings, and employs a deep learning architecture to predict both the occurrence and type of DDI events. Zhong et al. [2023] proposed DDI-GCN, a framework that uses graph convolutional networks (GCNs) to predict DDIs by analyzing drug chemical structures.

Zhu et al. [2024] used CNN and attention-based for sequence features and neural network, GAT, and SAGPooling for substructure features, combining them for DDI prediction. Asfand-E-Yar et al. [2024] employed a CNN model to predict DDIs. It integrated multiple drug features, including SMILES, enzymes, pathways, and targets, using four CNN sub-models, which are later concatenated for final prediction. Su and Qian [2024] used a neural network that predicts DDIs by combining drug structure and protein-binding features. It learns important patterns using graph-based learning. Peng and colleagues proposed a bi-directional network that integrated multi-head attention and intention mechanisms to fuse drug and protein features. It first encodes drugs using GCN and proteins using a combination of CNN and self-attention Peng et al. [2024]. Sun and colleagues utilized a GAT-based hierarchical dual-view representation learning network. Their network enables DDI prediction by capturing both local substructure interactions and global molecular information Sun and Zheng [2025].

3 Biotech-small molecule DDI Labels

Figure 3 represents four samples of positive labels in our dataset.

Figure 3: Four examples of different positive labels.

Table 1 demonstrates that there are 31 BSI labels with frequencies above 100. Therefore, we keep these as the number of positive types of labels, considering that the total number of existing BSIs is 43876.

Table 1: Distribution of the 96 positive labels used in the dataset, categorized by frequency range.

Count of labels	Frequency Range
3	> 4000
7	> 2000
10	> 1000
15	> 500
31	> 100
41	> 50

4 Metrics Analysis

The micro set of evaluation metrics treats all classes equally and focuses on the overall performance of the model. Furthermore, its preference is for the larger classes. The micro metrics, compares all true predictions (TP and TN) and all false predictions (FP and FN)of each class together. Therefore, it does not show the information at the class level. In other words, it does not care about how the model performs in individual classes. While Reporting micro metrics may show a high performance of the general model, it may have a very low performance for the classes with low frequency Sokolova and Lapalme [2009]. There are several evaluation metrics in this category and we choose some of them that are suitable for this work, i.e., accuracy, precision, recall, F1-Score, BACC, MCC, AUC and AUPR.

The macro set of evaluation metrics treats all classes equally, regardless of their size. Unlike micro-averaged metrics, macro metrics compute performance for each class separately based on its own samples and then average the results across all classes. Essentially, macro-evaluation means that the model's overall performance is the average of its performance on each class with any distribution. More precisely, the importance of each class does not depend on its size. For example, in medicine and disease diagnosis and prognosis, are considered just as important as common diseases. Furthermore, assigning equal weights to all classes ensures that the evaluation metrics are not dominated by larger classes Hinojosa Lee et al. [2024]. For this study, we select several macro-based evaluation metrics, including precision, recall, F1 score, AUC, and AUPR.

The weighted category lies between micro and macro evaluation. While it evaluates each class separately, like macro-averaging, it does not treat all classes equally. Instead, it assigns a weight to each class based on its size, meaning larger classes have a greater influence on the final score. More precisely, the overall evaluation is a weighted sum of the metrics computed for each class Hinojosa Lee et al. [2024]. For this study, we select several suitable weighted evaluation metrics, including precision, recall, F1 score, AUC, and AUPR.

5 Extra results

5.1 Confusion Matrix

Figure 4 shows the confusion matrix of the predicted labels against the true labels of 32 labels (31 positive labels plus the negative label). As the figure shows, most of the labels are predicted correctly. This confusion matrix is the basis for the following results.

Figure 4: Confusion matrix for biotech–small-molecule DDI prediction.

5.2 Micro

We have introduced micro results to evaluate the performance of the proposed method. Tables 2 and 3 report the results based on micro performance. The former provides accuracy, precision, recall, and F1-score; the latter presents MCC, AUROC, and AUPR. Regarding micro accuracy, BSI-Net achieves the highest performance, followed by the machine learning approaches, particularly random forest, which ranks next; the MLP method follows, while the CNN model ranks last with the lowest performance. Notably, machine learning approaches demonstrate high performance with lower complexity compared to the MLP and CNN models. The same pattern is observed for precision, recall, and F1-score in Table 2. Table 3 shows a similar pattern to the previous table. BSI-Net exhibits the best performance except in AUPR, where it ranks second, and random forest takes the top position. Again, this underscores the necessity for researchers to consider the strength of machine learning methods when proposing techniques. It is worth mentioning that the values in each row of Table 2 are equal. Therefore, the authors verified that all results are correct, and this observation is typical in micro value computations, making it less favorable for evaluating performance.

The observation that accuracy, precision, recall, and F1-score have identical values (including standard deviations) under micro-averaging is not a writing error—it is a mathematical property of micro-averaging in imbalanced multi-class settings. Under micro-averaging, accuracy, precision, recall, and F1-score yield identical values because micro-aggregation calculates metrics globally by counting total true positives (TP), false positives (FP), and false negatives (FN) across all classes. For a dataset with N samples, these metrics are computed as:

$$\text{Precision}_{\text{micro}} = \text{Recall}_{\text{micro}} = \text{F1}_{\text{micro}} = \text{Accuracy}_{\text{micro}} = \frac{\sum \text{TP}}{\sum \text{TP} + \sum \text{FP}}$$

This equality occurs because micro-averaging treats every prediction equally, regardless of class imbalance. Thus, the standard deviations (reflecting variability across cross-validation folds) are also identical for these metrics. Note that MCC and AUC/PR remain distinct as they are computed differently. Micro-averaging pools all predictions into a single confusion matrix, making precision = recall = F1 = accuracy when averaged this way Grandini et al. [2020], Sokolova and Lapalme [2009].

Table 4 shows BSI-Net performs well across all evaluation methods. The nearly equal micro scores (Se=0.986, Sp=0.983) mean it works consistently for all drug pairs. The higher macro specificity (0.9995) proves it's especially good at avoiding false alarms, while the weighted scores (0.981-0.997) confirm this works for both common and rare interactions.

Table 2: Evaluation of Methods in Micro Regime (first part).

Methods	Accuracy(std)	Precision(std)	Recall(std)	F1-Score(std)
**SVM**	0.9764(0.001)	0.9764(0.001)	0.9764(0.001)	0.9764(0.001)
**RF**	0.9827(0.001)	0.9827(0.001)	0.9827(0.001)	0.9827(0.001)
**XGBoost**	0.9730(0.002)	0.9730(0.002)	0.9730(0.002)	0.9730(0.002)
**MLP**	0.7381(0.014)	0.7381(0.014)	0.7381(0.014)	0.7381(0.014)
**CNN**	0.6048(0.048)	0.6048(0.048)	0.6048(0.048)	0.6048(0.048)
**BSI-Net**	**0.9861(0.009)**	**0.9861(0.009)**	**0.9861(0.009)**	**0.9861(0.009)**

Table 3: Evaluation of Methods in Micro regime (second part).

Methods	MCC(std)	AUROC(std)	AUPR(std)
**SVM**	0.9675(0.002))	0.9997(0.000)	0.9946(0.000)
**RF**	0.9761(0.002)	0.9997(0.000)	**0.9974(0.000)**
**XGBoost**	0.9625(0.002)	0.9998(0.000)	0.9953(0.001)
**MLP**	0.6941(0.013)	0.9877(0.001)	0.7980(0.014)
**CNN**	0.5629(0.040)	0.9781(0.004)	0.6621(0.053)
**BSI-Net**	**0.9809(0.012)**	**1.0000(0.000)**	0.9908(0.009)

Table 4: Performance comparison of BSI-Net across evaluation regimes: Micro-averaged, Macro-averaged, and Weighted Sensitivity (Se) and Specificity (Sp) scores.

Method	Micro	Macro	Weighted
BSI-Net	Se: 0.9861<br>Sp: 0.9832	Se: 0.9635<br>Sp: 0.9995	Se: 0.9814<br>Sp: 0.9978

5.3 Comparison

Figure 5 compares BSI-Net's AUROC and AUPR in weighted mode with state-of-the-art methods, though this comparison is not entirely fair due to dataset differences. Multi-SBI Huang et al. [2022] only reported binary results, but BSI-Net outperforms all compared methods, with random forest performing second best.

Figure 5: Comparison of BSI-Net with other methods based on F1-score and AUPR.

To ensure a fair evaluation, we re-implemented the CB-TIP method using the same experimental settings and applied it to our newly constructed dataset. Table 5 presents the comparative results between CB-TIP and our proposed method, BSI-Net. As demonstrated, BSI-Net outperforms CB-TIP across all performance metrics, including Precision, Recall, F1-score, AUROC, and AUPRC. These results highlight the effectiveness and robustness of BSI-Net and validate its superior predictive capability under consistent evaluation conditions. The fundamental differences in the input data are also noteworthy. In our proposed method, more advanced techniques have been used for similarity assessment and feature vector generation. Additionally, the structural matrices we created to represent drugs are completely absent in the compared methods, but we used those matrices for utilizing all our features.

Table 5: BSI-Net vs. CB-TIP performance in weighted biotech–small-molecule DDI prediction.

**Method**	**Precision (std)**	**Recall (std)**	**F1-score (std)**	**AUROC (std)**	**AUPRC (std)**
CB-TIP	0.9574 (0.047)	0.9557 (0.075)	0.9546 (0.058)	0.9990 (0.010)	0.9880 (0.280)
BSI-Net	**0.9866** (0.022)	**0.9861** (0.025)	**0.9861** (0.021)	**0.9999** (0.000)	**0.9981** (0.007)

6 Ablation Study

6.1 Parameter Tuning and Enhancements

To ensure robust and accurate classification performance, all models were individually fine-tuned through extensive hyperparameter exploration. For deep learning

approaches—BSI-Net (based on a graph attention network), CNN, and multi-layer perceptron (MLP)—a range of configurations were tested, including variations in learning rate, dropout rate, activation functions, and optimization algorithms. The optimal settings were selected based on validation performance, with all three models achieving their best results using the Adam optimizer and ReLU activation function but differing in dropout rates and architectural structure. These final hyperparameter settings for the deep learning models are summarized in Table 6.

Table 6: Best hyperparameter settings for deep learning models.

**BSI-Net**	**CNN**	**MLP**
LR: 0.001<br>Optimizer: Adam<br>Activation: ReLU<br>Dropout: 0.2<br>Output: Softmax	LR: 0.001<br>Optimizer: Adam<br>Activation: ReLU<br>Dropout: 0.5<br>Output: Softmax	LR: 0.001<br>Optimizer: Adam<br>Activation: ReLU<br>Dropout: 0.3<br>Output: Softmax

Similarly, traditional machine learning models were tuned using grid search across relevant hyperparameters. The support vector machine (SVM) achieved optimal performance with a linear kernel and a regularization parameter of $C = 10$. The best configuration for the random forest (RF) classifier included 10 estimators, the `log_loss` splitting criterion, and the `log2` method for feature selection. XGBoost was fine-tuned by varying the number of estimators, tree depth, and sampling ratios, resulting in the optimal combination of 100 estimators, a maximum depth of 3, a learning rate of 0.1, and both subsample and colsample_bytree set to 0.5. These optimized hyperparameters for the classical models are detailed in Table 7, and were used consistently throughout the evaluation process.

Table 7: Best hyperparameter settings for traditional machine learning models.

**SVM**	**RF**	**XGBoost**
Kernel: Linear<br>C: 10	n_estimators: 10<br>Criterion: log_loss<br>Max Features: log2	n_estimators: 100<br>Max Depth: 3<br>LR: 0.1<br>Subsample: 0.5<br>Colsample_bytree: 0.5

To ensure BSI-Net performed at its best, we ran thorough tests adjusting all its key settings. We experimented with over 1,500 different combinations, running each multiple times to get reliable results. Through this process, we found the sweet spot for each component. The graph attention layers worked best with [128,256] hidden dimensions - smaller sizes like [64,64] couldn't capture enough

patterns, while larger ones like [256,512] started memorizing the training data instead of learning properly. For the final prediction layers, a deeper [1024,512] structure outperformed simpler versions because it could handle more complex relationships in the data. We discovered that using a moderate dropout rate of 0.2 gave the best balance - too little (below 0.15) led to overfitting, while too much (above 0.25) caused the model to ignore useful information. The learning rate of 0.001 turned out to be ideal, training the model efficiently without causing instability - slower rates below 0.0005 took nearly twice as long to converge. When it came to batch size, 32 proved optimal, large enough to be stable but small enough to maintain good gradient diversity, unlike larger batches of 128 which hurt performance. After identifying these optimal settings through careful testing, we validated them using 10-fold cross-validation to ensure they worked consistently across different data splits. This comprehensive tuning was crucial - using suboptimal settings could have reduced performance by up to 7.4%. The final configuration delivers BSI-Net's best possible balance of accuracy and reliability.

6.2 Contribution of Different Components

A comprehensive ablation study was performed to evaluate the contribution of each component of the proposed BSI-Net model. The analysis involved comparisons across various model configurations to understand how each input and module affects prediction performance.

We began by evaluating two baseline models, both using only similarity matrices: a simple multilayer perceptron (MLP) and an MLP enhanced with ProtBert embeddings, but without the graph attention mechanism. As seen in Tables 8,9, and10, these configurations performed significantly worse than BSI-Net, with F1-scores around 0.70–0.73 (micro), 0.63–0.65 (macro), and 0.73–0.76 (weighted), highlighting the limitations of shallow architectures and the need for more expressive modeling.

To assess the importance of each main input, we conducted two key experiments: (1) removing similarity matrices while keeping SMILES and ProtBert features, and (2) removing ProtBert while keeping similarity matrices and SMILES features. Removing the similarity matrices (GAT + MLP with SMILES and ProtBert only) led to the most substantial drop in performance: the micro AUPR dropped from 0.9908 (full BSI-Net) to 0.4056, and the macro F1-score fell drastically to 0.3104. This result demonstrates that similarity matrices are critical for capturing pharmacological relationships and for guiding effective learning.

On the other hand, removing ProtBert while keeping SMILES and similarity matrices (GAT + MLP without ProtBert) also reduced performance, though to a lesser extent. The model still achieved a relatively high micro F1-score of 0.9716 and macro F1 of 0.9192, indicating that while ProtBert adds value, the similarity

matrices and SMILES structures already capture much of the necessary information.

To better understand the role of similarity matrices, we gradually enriched them. Starting from features such as Tanimoto similarity and sequence alignment, we then incorporated drug–target interactions, and finally added DDIs. Each addition led to measurable performance gains.

Finally, we examined a model that excluded SMILES representations entirely and used only similarity matrices and ProtBert embeddings (MLP with similarity matrices + ProtBert, without GAT). This setup underperformed significantly (e.g., macro F1 of 0.6307, weighted F1 of 0.7366), confirming that the molecular structure of small-molecule drugs is also essential and contributes complementary information.

In conclusion, this ablation study demonstrates that all three components—similarity matrices, ProtBert embeddings, and SMILES-based molecular graphs—are indispensable for optimal performance. Their integration in BSI-Net leads to a well-rounded model that significantly outperforms all ablated variants, achieving the highest results across all metrics (e.g., micro F1-score of 0.9861, macro F1-score of 0.9627, and weighted F1-score of 0.9861). These findings validate the design choices of BSI-Net and offer insights for constructing robust DDI prediction models.

Table 8: Micro results in ablation study

Method	F1-Score(std)	MCC(std)	AUROC(std)	AUPR(std)
MLP with Similarity Matrices	0.7381(0.014)	0.6941(0.013)	0.9877(0.001)	0.7980(0.014)
MLP with Similarity Matrices + ProtBert (without GAT)	0.7037(0.0277)	0.6606(0.024)	0.9824(0.004)	0.7422(0.033)
GAT + MLP without ProtBERT	0.9716(0.008)	0.9608(0.012)	0.9999(0.001)	0.9707(0.016)
GAT + MLP without Similarity Matrices (SMILES + ProtBert)	0.7259(020)	0.6047(0.028)	0.9865(0.002)	0.4056(0.028)
**BSI-Net**	**0.9861(0.009)**	**0.9809(0.012)**	**1.0000(0.000)**	**0.9908(0.009)**

Table 9: Macro results in ablation study

Method	Precision(std)	Recall(std)	F1-Score(std)	AUROC(std)	AUPR(std)
MLP with Similarity Matrices	0.5632(0.020)	0.9228(0.004)	0.6551(0.018)	0.9933(0.001)	0.9040(0.005)
MLP with Similarity Matrices + ProtBert (without GAT)	0.5449(0.026)	0.9142(0.008)	0.6307(0.026)	0.9920(0.001)	0.8842(0.010)
GAT + MLP without ProtBERT	0.9400(0.069)	0.9153(0.136)	0.9192(0.101)	0.9996(0.008)	0.9707(0.065)
GAT + MLP without Similarity Matrices (SMILES + ProtBert)	0.4866(0.384)	0.3037(0.327)	0.3104(0.312)	0.9612(0.029)	0.4056(0.305)
**BSI-Net**	**0.9705(0.053)**	**0.9609(0.073)**	**0.9627(0.058)**	**0.9999(0.000)**	**0.9908(0.026)**

Table 10: Weighted results in ablation study

Method	Precision(std)	Recall(std)	F1-Score(std)	AUROC(std)	AUPR(std)
MLP with Similarity Matrices	0.8592(0.007)	0.7381(0.014)	0.7655(0.014)	0.9788(0.002)	0.9294(0.006)
MLP with Similarity Matrices + ProtBert (without GAT)	0.8515(0.008)	0.7037(0.027)	0.7366(0.025)	0.9743(0.003)	0.9166(0.008)
GAT + MLP without ProtBERT	0.9724(0.035)	0.9716(0.050)	0.9711(0.040)	0.9995(0.005)	0.9939(0.020)
GAT + MLP without Similarity Matrices (SMILES + ProtBert)	0.7053(0.212)	0.7259(0.343)	0.6891(0.285)	0.9469(0.029)	0.7465(0.256)
**BSI-Net**	**0.9866(0.022)**	**0.9861(0.025)**	**0.9861(0.021)**	**0.9999(0.000)**	**0.9981(0.007)**

7 Predicted Labels

Table 12 presents a selected subset of biotech–small-molecule drug pairs, showing both their known interaction labels and the corresponding labels predicted by BSI-Net. To aid interpretation, table 11 provides detailed descriptions of the drug-drug interaction (DDI) classes predicted by BSI-Net that are also represented in Table 12.

Table 11: Explanation for each class number

Class Number	Class Description
1	The metabolism of drug2 can be increased when combined with drug1.
2	The risk or severity of adverse effects can be increased when drug2 is combined with drug1.
4	The therapeutic efficacy of drug1 can be decreased when used in combination with drug2.
5	The risk or severity of hypoglycemia can be increased when drug2 is combined with drug1.
8	The metabolism of drug2 can be decreased when combined with drug1.
17	The risk or severity of Thrombosis can be increased when drug2 is combined with drug1.
24	The risk or severity of neutropenia can be increased when drug1 is combined with drug2.
27	The serum concentration of drug2 can be decreased when it is combined with drug1.

Table 12: Biotech–Small-molecule Drug Pairs with True and Predicted Labels

**Biotech Drug**	**Small-molecule Drug**	**True Label**	**Predicted Label**
Insulin_human	Praziquantel	0	1
Darbepoetin_alfa	Cilazapril	0	27
Erythropoietin	Finafloxacin	0	17
Darbepoetin_alfa	Perindopril	0	27
Insulin_human	Trofinetide	0	5
Insulin_human	Diclofenac	0	1
Darbepoetin_alfa	Enalaprilat	0	27
Insulin_human	Pirfenidone	0	1
Darbepoetin_alfa	Trandolapril	0	27
Anakinra	Fosnetupitant	0	1
Insulin_human	Primaquine	0	1
Darbepoetin_alfa	Quinapril	0	27
Anakinra	Tivozanib	0	1
Darbepoetin_alfa	Cycloguanil	0	17
Darbepoetin_alfa	Moexipril	0	27
Erythropoietin	Cinnamaldehyde	0	17
Darbepoetin_alfa	Chlortetracycline	0	17
Darbepoetin_alfa	Ramipril	0	27
Insulin_human	Leflunomide	0	1
Darbepoetin_alfa	Spirapril	0	27
Sargramostim	Topotecan	0	24
Anakinra	Gepirone	0	1
Darbepoetin_alfa	Tafenoquine	0	17
Insulin_human	Binimetinib	0	1
Insulin_human	Aliskiren	0	5
Anakinra	Bezafibrate	0	1
Darbepoetin_alfa	Fosinopril	0	27
Insulin_human	Levobupivacaine	0	1
Darbepoetin_alfa	Artemotil	0	17
Insulin_human	Clopidogrel	0	1
Darbepoetin_alfa	Azithromycin	0	17
Urokinase	Nitroglycerin	0	4
Anistreplase	Nitroglycerin	0	4
Insulin_human	Zileuton	0	1
Erythropoietin	Chlortetracycline	0	17
Interferon_alfa-n1	Telbivudine	0	2
Darbepoetin_alfa	Rescinnamine	0	27
Darbepoetin_alfa	Lefamulin	0	17
Insulin_human	Dacarbazine	0	1
Interferon_alfa-n1	Dosulepin	0	8
Anakinra	Prednisolone_phosphate	0	1
Etanercept	Levomilnacipran	0	1
Anakinra	Lumacaftor	0	1
Erythropoietin	Chloramphenicol	0	17

References

M. H. Al-Rabeah and A. Lakizadeh. Prediction of drug-drug interaction events using graph neural networks based feature extraction. *Scientific Reports*, 12(1): 15590, 2022.

M. Asfand-E-Yar, Q. Hashir, A. A. Shah, H. A. M. Malik, A. Alourani, and W. Khalil. Multimodal cnn-ddi: using multimodal cnn for drug to drug interaction associated events. *Scientific Reports*, 14(1):4076, 2024.

Y. Chen, T. Ma, X. Yang, J. Wang, B. Song, and X. Zeng. Muffin: multi-scale feature fusion for drug–drug interaction prediction. *Bioinformatics*, 37(17): 2651–2658, 2021.

M. Grandini, E. Bagli, and G. Visani. Metrics for multi-class classification: an overview. *arXiv preprint arXiv:2008.05756*, 2020.

K. Han, P. Cao, Y. Wang, F. Xie, J. Ma, M. Yu, J. Wang, Y. Xu, Y. Zhang, and J. Wan. A review of approaches for predicting drug–drug interactions based on machine learning. *Frontiers in pharmacology*, 12:814858, 2022.

M. C. Hinojosa Lee, J. Braet, and J. Springael. Performance metrics for multilabel emotion classification: comparing micro, macro, and weighted f1-scores. *Applied Sciences*, 14(21):9863, 2024.

D. Huang, H. He, J. Ouyang, C. Zhao, X. Dong, and J. Xie. Small molecule drug and biotech drug interaction prediction based on multi-modal representation learning. *BMC bioinformatics*, 23(1):561, 2022.

S. Lin, Y. Wang, L. Zhang, Y. Chu, Y. Liu, Y. Fang, M. Jiang, Q. Wang, B. Zhao, Y. Xiong, et al. Mdf-sa-ddi: predicting drug–drug interaction events based on multi-source drug fusion, multi-source feature fusion and transformer self-attention mechanism. *Briefings in Bioinformatics*, 23(1):bbab421, 2022.

L. Peng, X. Liu, L. Yang, L. Liu, Z. Bai, M. Chen, X. Lu, and L. Nie. Bindti: a bi-directional intention network for drug-target interaction identification based on attention mechanisms. *IEEE Journal of Biomedical and Health Informatics*, 2024.

J. Y. Ryu, H. U. Kim, and S. Y. Lee. Deep learning improves prediction of drug–drug and drug–food interactions. *Proceedings of the national academy of sciences*, 115(18):E4304–E4311, 2018.

M. Sokolova and G. Lapalme. A systematic analysis of performance measures for classification tasks. *Information processing & management*, 45(4):427–437, 2009.

J. Su and Y. Qian. Ddi-transform: A neural network for predicting drug-drug interaction events. *Quantitative Biology*, 12(2):155–163, 2024.

J. Sun and H. Zheng. Hdn-ddi: a novel framework for predicting drug-drug interactions using hierarchical molecular graphs and enhanced dual-view representation learning. *BMC bioinformatics*, 26(1):28, 2025.

D. S. Wishart, Y. D. Feunang, A. C. Guo, E. J. Lo, A. Marcu, J. R. Grant, T. Sajed, D. Johnson, C. Li, Z. Sayeeda, et al. Drugbank 5.0: a major update to the drugbank database for 2018. *Nucleic acids research*, 46(D1):D1074–D1082, 2018.

C. Yan, G. Duan, Y. Zhang, F.-X. Wu, Y. Pan, and J. Wang. Predicting drug-drug interactions based on integrated similarity and semi-supervised learning. *IEEE/ACM transactions on computational biology and bioinformatics*, 19(1): 168–179, 2020.

H. Yu, K.-T. Mao, J.-Y. Shi, H. Huang, Z. Chen, K. Dong, and S.-M. Yiu. Predicting and understanding comprehensive drug-drug interactions via semi-nonnegative matrix factorization. *BMC systems biology*, 12:101–110, 2018.

H. Yu, W. Dong, and J. Shi. Raneddi: Relation-aware network embedding for drug-drug interaction prediction. *Information Sciences*, 582:167–180, 2022.

W. Zhang, Y. Chen, F. Liu, F. Luo, G. Tian, and X. Li. Predicting potential drug-drug interactions by integrating chemical, biological, phenotypic and network data. *BMC bioinformatics*, 18:1–12, 2017.

Y. Zhong, H. Zheng, X. Chen, Y. Zhao, T. Gao, H. Dong, H. Luo, and Z. Weng. Ddi-gcn: Drug-drug interaction prediction via explainable graph convolutional networks. *Artificial Intelligence in Medicine*, 144:102640, 2023.

J. Zhu, Y. Liu, C. Wen, and X. Wu. Dgdfs: Dependence guided discriminative feature selection for predicting adverse drug-drug interaction. *IEEE Transactions on Knowledge and Data Engineering*, 34(1):271–285, 2020.

J. Zhu, C. Che, H. Jiang, J. Xu, J. Yin, and Z. Zhong. Ssf-ddi: a deep learning method utilizing drug sequence and substructure features for drug–drug interaction prediction. *BMC bioinformatics*, 25(1):39, 2024.
